# Why Do Men Accumulate Abdominal Visceral Fat?

**DOI:** 10.3389/fphys.2019.01486

**Published:** 2019-12-05

**Authors:** Andromeda M. Nauli, Sahar Matin

**Affiliations:** ^1^Department of Pharmaceutical Sciences, College of Pharmacy, Marshall B. Ketchum University, Fullerton, CA, United States; ^2^College of Pharmacy, Marshall B. Ketchum University, Fullerton, CA, United States

**Keywords:** visceral, adipocyte, gender, lipoprotein, chylomicron, intestine, absorption, belly

## Abstract

Men have a higher tendency to accumulate abdominal visceral fat compared to pre-menopausal women. The accumulation of abdominal visceral fat in men, which is a strong independent predictor of mortality, is mainly due to the higher dietary fat uptake by their abdominal visceral fat. Since dietary fat is absorbed by the enterocytes and transported to the circulation in the forms of chylomicrons and very low density lipoproteins (VLDLs), it is crucial to understand how these lipoproteins are different between men and women. The chylomicrons in men are generally bigger in size and more in quantity than those in women. During the postprandial state, these chylomicrons congest the lamina propria and the low-pressure lymphatics. In this paper, we propose that this congestion predisposes the chylomicron triglycerides to hydrolysis by lipoprotein lipase (LPL). The liberated fatty acids are then stored by the nearby abdominal visceral adipocytes, leading to the accumulation of abdominal visceral fat. These mechanisms perhaps explain why men, through their bigger and higher production of chylomicrons, are more likely to accumulate abdominal visceral fat than pre-menopausal women. This accumulation eventually leads to belly enlargement, which confers men their apple-shaped body.

## Body Fat

The sex differences in body composition have been well established (Karastergiou et al., 2012; Palmer and Clegg, 2015). Women have a higher body fat percentage than men, and men have a higher muscle mass percentage than women. As the BMI increases in both sexes, the body fat percentage remains higher in women than men (Schorr et al., 2018). Importantly, the differences between the two sexes are not only in the percentage of total body fat but also in its distribution to the different parts of the body (Grauer et al., 1984).

### Types of Body Fat

Body fat can be classified into brown, beige, and white fat (Harms and Seale, 2013). In terms of relative abundance in mitochondria, brown fat is the most abundant and white fat is the least. Unlike brown and beige fat, white fat is not capable of thermogenesis. Note that body fat in adult humans consists of mostly white fat.

Based on its location in the body, white fat can be further subcategorized into subcutaneous, visceral, and ectopic fat. Ectopic fat, which is the least in quantity, is located within the internal organs. Intrahepatocellular fat, intrapancreatic fat, intramyocellular fat, and intracardiomyocellular fat are all considered as ectopic fat. The fat that surrounds the internal organs is generally considered as visceral fat. The epicardial fat and the abdominal visceral fat surround the myocardia and gastrointestinal organs, respectively, and are both considered as visceral fat (Bertaso et al., 2013; Frank et al., 2018). Subcutaneous fat, which is more abundant in women (Karastergiou et al., 2012), is located throughout the layer deep to the skin (hypodermis).

As visceral fat in the abdomen accumulates, the belly becomes visibly bigger—a phenomenon that is commonly known as belly fat development. Of note, belly fat consists of not only abdominal visceral fat but also abdominal subcutaneous fat. Although waist circumference correlates strongly with total belly fat, it does not correlate as strongly with abdominal visceral fat (Grundy et al., 2013). Furthermore, the correlation of waist circumference with abdominal visceral fat is weaker in women than in men. Therefore, the inference of the amount of abdominal visceral fat from waist circumference should be made cautiously, especially in women.

### Abdominal Visceral Fat

**FIGURE 1:**
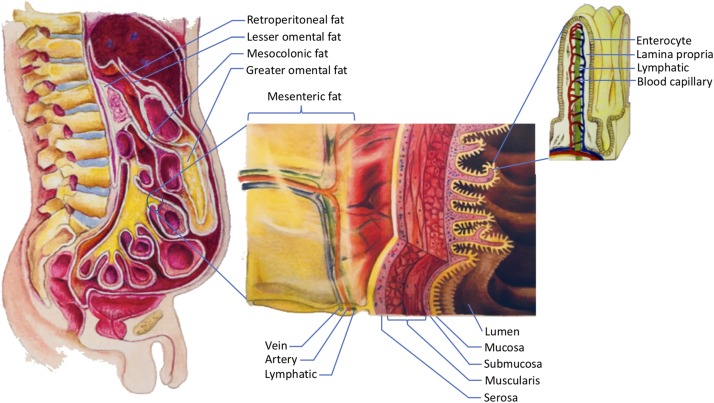
The storage of dietary triglycerides by abdominal visceral fat. Dietary triglycerides are digested and taken up by the enterocytes that line the intestinal lumen. The enterocytes secrete the dietary triglycerides in the form of VLDLs and chylomicrons to the lamina propria. Within the lamina propria, there are numerous blood capillaries (red/blue) and lymphatic capillaries (green). Due to their smaller size, some of the VLDLs can readily enter the lumen of the blood capillaries. In contrast, chylomicrons that are produced more by males are more likely to be retained in the lamina propria as they attempt to gain access to the lumen of the lymphatics. The higher retention of chylomicrons in the lamina propria predisposes their triglycerides to LPL hydrolysis. The liberated fatty acids, which are the products of LPL hydrolysis, can then be delivered to the abdominal visceral adipocytes that are located within the retroperitoneum and mesenteries. The fat that lies within the retroperitoneum is called retroperitoneal fat, and the fat in the mesenteries is known as intraperitoneal fat. The intraperitoneal fat depots shown here are mesocolonic, lesser omental, greater omental, and mesenteric fat. Note that the liberated fatty acids supply the abdominal visceral adipocytes prior to the subcutaneous adipocytes. VLDL = very low density lipoprotein; LPL = lipoprotein lipase.

This paper focuses specifically on the visceral fat in the abdomen. In order to understand abdominal visceral fat, a closer look at the anatomy of mesenteries and retroperitoneum is warranted (see Figure 1). Mesenteries connect the gastrointestinal organs that are located within the abdominal cavity to the wall of the abdominal cavity. Most of the connections are made to the posterior rather than the anterior wall of the abdominal cavity. As such, organs that reside within the posterior wall of the abdominal cavity do not have any mesenteries. These organs are commonly known as retroperitoneal organs. The two retroperitoneal organs depicted in Figure 1 are pancreas and duodenum. The fat that surrounds these retroperitoneal organs is known as retroperitoneal fat. Note that other retroperitoneal organs, such as kidneys, ascending colon, and descending colon, are not shown in Figure 1.

In addition to adhering the gastrointestinal organs to their abdominal wall, mesenteries protect numerous nerves, blood vessels, and lymphatic vessels of the gastrointestinal system. Importantly, mesenteries are also capable of storing a significant amount of fat. The greater omentum, lesser omentum, mesentery proper, and mesocolon are examples of mesenteries. As indicated in Figure 1, the fat that is located within these mesenteries is known as the greater omental fat, lesser omental fat, mesenteric fat, and mesocolonic fat, respectively. The fat in these mesenteries is collectively referred to as intraperitoneal fat.

Both intraperitoneal fat and retroperitoneal fat constitute abdominal visceral fat, which explains why many investigators include retroperitoneal fat when measuring abdominal visceral fat (Hung et al., 2014). There are several reasons to consider the retroperitoneal fat as part of the abdominal visceral fat. First, retroperitoneal fat surrounds retroperitoneal organs. Thus, it should be categorized as visceral instead of ectopic or subcutaneous fat. Second, the lymph fluid of the gastrointestinal tract drains through the smaller lymphatic vessels within the mesenteries before entering the larger lymphatics. The larger lymphatics, such as cisterna chyli, are retroperitoneal. Consequently, the adipocytes that are present in the mesenteries and retroperitoneum receive the same supply of lipid-rich chyle. This chyle will eventually be drained into the systemic blood circulation before supplying the subcutaneous fat. Hence, from the nutrient supply perspective, retroperitoneal fat is more similar to intraperitoneal fat than subcutaneous fat. For further discussions on the anatomy of the gastrointestinal circulation, please refer to our previously published paper (Nauli and Nauli, 2013). Third, unlike subcutaneous fat, both retroperitoneal and intraperitoneal fat increase the risk of metabolic syndrome (Hung et al., 2014). Therefore, it is conceivable that abdominal visceral fat should include both intraperitoneal and retroperitoneal fat.

**FIGURE 2:**
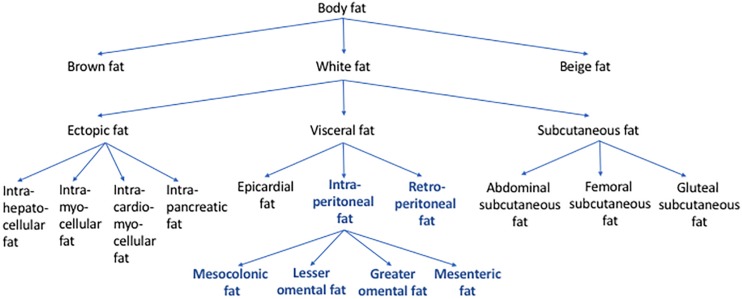
Types of body fat in relation to abdominal visceral fat. Body fat can be categorized into brown, beige, and white fat. Based on its location, white fat can be further categorized into ectopic, subcutaneous, and visceral fat. Some of the examples of ectopic fat are intrahepatocellular, intrapancreatic, intramyocellular, and intracardiomyocellular fat. Subcutaneous fat includes abdominal, femoral, and gluteal subcutaneous fat. Visceral fat includes epicardial, retroperitoneal, and intraperitoneal fat. The intraperitoneal fat can be further subcategorized into mesocolonic, lesser omental, greater omental, and mesenteric fat. Abdominal visceral fat includes both retroperitoneal and intraperitoneal fat (bold/blue).

The fact that abdominal visceral fat is often simply referred to as “visceral fat” can sometimes cause confusion. When the amount of “visceral fat” is measured from the abdominal region, it is arguably more appropriate to label it as abdominal visceral fat instead of “visceral fat.” As mentioned above, visceral fat includes not only the abdominal visceral fat but also the fat depots that surround other non-abdominal organs, such as the epicardial fat. Figure 2 shows how different types of body fat relate to abdominal visceral fat.

### Women Are Shaped Like a Pear and Men Like an Apple

Due to their tendency of accumulating abdominal visceral fat (Grauer et al., 1984), men are more likely to develop an apple-shaped body. The excessive accumulation of abdominal visceral fat is also known as android obesity. In contrast, the pear-shaped is often ascribed to pre-menopausal women because of their tendency of accumulating subcutaneous fat in the thigh (femoral) and buttock (gluteal) regions (Karastergiou et al., 2012). The obesity resulted from the predominant subcutaneous fat accumulation is also known as gynoid obesity.

A common misconception is that beer consumption can specifically lead to belly fat accumulation. This misconception gave rise to the term “beer belly.” Studies have shown that beer consumption does not specifically increase the belly size but rather increases the overall body weight (Schutze et al., 2009). Therefore, it is unlikely that beer consumption specifically increases the abdominal visceral fat or is particularly responsible for android obesity.

### Abdominal Visceral Fat Is Associated Strongly With Metabolic Abnormalities

Although the abdominal subcutaneous fat and the intrahepatocellular fat are associated with a higher risk of mortality in men, only the abdominal visceral fat is a strong independent predictor of mortality in men (Kuk et al., 2006). The association of abdominal visceral fat with mortality is not unique to men as abdominal visceral fat is also a strong predictor of mortality in obese women (Koster et al., 2015). Consequently, it is important to understand the pathogenesis of abdominal visceral fat and its association with metabolic complications.

Obesity can impede the functions of microvasculature. Studies in male hamsters have revealed that android obesity is associated not only with insulin resistance but also a diminution of capillary density and an increase in macromolecular permeability (Costa et al., 2011). This microvascular dysfunction may eventually lead to the development of hypertension (Covassin et al., 2018), which is one of the criteria of metabolic syndrome. Interestingly, the male hamsters that were subjected to the high-fat diet in these studies accumulated fat almost exclusively in the abdominal visceral region with minimal fat accumulation in the subcutaneous region. This important observation suggests that the high intake of dietary fat in men promotes fat accumulation that is rather specific to the abdominal visceral depot. The increase in vascular permeability in the android obesity described above (Costa et al., 2011) also suggests that fat accumulation in the abdominal visceral depot is caused by the dysregulation of the vascular endothelial cells. In this regard, the endothelial cells that line the affected vasculatures may be a key contributor in the development of android obesity as suggested in a recent proposed two-way communication hypothesis of vascular dysfunction in obesity (Graupera and Claret, 2018).

We have previously described how android obesity may lead to insulin resistance (Nauli, 2012). As the abdominal visceral fat accumulates, macrophages infiltration increases (Xu et al., 2003). The infiltrating macrophages are known to release inflammatory cytokines. These cytokines, which include TNFα, are capable of causing the surrounding abdominal visceral adipocytes to become insulin resistant and liberate their fatty acids (Samuel and Shulman, 2016). This flux of fatty acids is detrimental to the liver and pancreas (Matsuzawa et al., 1995). Note that the flux of fatty acids to the liver also occurs after a bolus feeding of triglycerides. Bolus feeding of fat has been shown to increase unesterified fatty acid concentration in the portal vein (Kristensen et al., 2006). Therefore, it is possible that both the abdominal visceral fat and the frequent consumption of high-fat diet contribute to the pathogenesis of metabolic syndrome through the increased flux of fatty acids to the portal venous circulation.

Perhaps it is worthwhile to examine the results of the omentectomy studies. One of the earliest omentectomy studies shows that patients who received omentectomy and adjustable gastric banding had a better metabolic profile than those with the adjustable gastric banding alone (Thorne et al., 2002). The patients with omentectomy and adjustable gastric banding lost more weight than the patients with adjustable gastric banding alone, albeit not significantly. Studies by Dillard et al. (2013) similarly show that omentectomy improved the metabolic profile without causing significantly more weight loss than the control group. On the contrary, studies by Fabbrini et al. (2010) show that omentectomy did not improve the metabolic profile when the amount of weight loss was matched between the omentectomy and the control groups. Several other studies did not also show any significant metabolic improvements in the omentectomy group (Csendes et al., 2009; Sdralis et al., 2013; Andersson et al., 2017). The conflicting results reported from these omentectomy studies could be due to several factors: the limited number of the participants, the pre-existing metabolic conditions of the participants, the procedures during surgery, the time intervals selected to assess the metabolic outcome of the omentectomy, and/or the participants’ dietary lifestyle.

Based on the discussion above, it can be concluded that more studies are needed to elucidate the exact roles of the abdominal visceral fat in the pathogenesis of metabolic syndrome. It is clear, however, that abdominal visceral fat is associated with many detrimental effects (Booth et al., 2014; Santosa and Jensen, 2015).

## Men’s Gut Favors Abdominal Visceral Fat Accumulation

The underlying mechanisms of why men are more likely than pre-menopausal women to accumulate abdominal visceral fat remain unclear. Evidence indicates that once dietary fat is absorbed by the gut, the intestinal lipoproteins produced between males and females are not identical (Vahouny et al., 1980). How these intestinal lipoproteins may contribute to the sex differences in the regional body fat distribution will be discussed below.

### More Uptake of Fat by Men’s Abdominal Visceral Fat Depot

Accumulation of fat is the result of a higher calorie intake relative to the energy expenditure. From the adipocyte perspective, this corresponds to more uptake of nutrients than the breakdown of fat by adipocytes. The fat catabolism of adipocytes, also known as lipolysis, is mediated partly by epinephrine. Upon binding of epinephrine to β adrenergic receptors, lipolysis is stimulated. On the contrary, the binding of epinephrine to α2A adrenergic receptors results in the inhibition of lipolysis (Richelsen, 1986). In essence, β receptors are lipolytic and α2A receptors are anti-lipolytic.

Studies have shown that estrogen reduces the lipolysis in the gluteal subcutaneous adipocytes (Gavin et al., 2013). The reduced lipolysis in the gluteal subcutaneous adipocytes in women is likely due to estrogen receptor α-mediated increase in α2A receptors. The estrogen-stimulated increase of these anti-lipolytic receptors in the subcutaneous adipocytes, but not in the abdominal visceral adipocytes, may contribute to the more pronounced lipolysis in the abdominal visceral adipocytes relative to the subcutaneous adipocytes in women (Pedersen et al., 2004).

The net fat accumulation in a particular fat depot, however, depends not only on its adipocyte lipolysis but also on the nutrient uptake of its adipocytes as well as its total number of adipocytes. Since estrogen is capable of stimulating human pre-adipocyte proliferation (Anderson et al., 2001), the reduced gluteal subcutaneous adipocyte lipolysis may not necessarily lead to an overall reduction in the lipolysis of the gluteal subcutaneous fat depot. In fact, studies comparing the lipolysis and nutrient uptake of various fat depots indicate that women have more lipolysis than men in the lower body fat depot, whereas men have more lipolysis than women in the abdominal visceral fat depot (Santosa and Jensen, 2008). The studies suggest that relative to lipolysis, fat uptake contributes more significantly to sex differences in body fat distribution. In other words, women accumulate more fat in the subcutaneous depot primarily because that depot takes up more fat in women than men. Likewise, men accumulate more fat in the abdominal visceral depot because their fat depot takes up more fat than women.

Some factors contributing to the tendency of non-obese women to accumulate subcutaneous fat include their high LPL activities in subcutaneous fat depots (Arner et al., 1991) and their high catabolic rate of hepatic-derived lipoproteins (Matthan et al., 2008). LPL activities are critical for body fat accumulation (Serra et al., 2017) because most of the fatty acids that are taken up by the fat depots are derived from the hydrolysis of lipoprotein triglycerides (Weinstock et al., 1997). The high LPL activities in the lower part of women’s body are evident in both preprandial and postprandial state (Votruba and Jensen, 2006). A recent study shows that testosterone is capable of suppressing the LPL activity and fat storage in the femoral region (Santosa et al., 2017). Furthermore, it shows a significant correlation between the LPL activity and the fat storage, implying that women’s tendency to accumulate fat in their subcutaneous depots is due to their high subcutaneous fat LPL activities. Another factor that promotes subcutaneous fat accumulation in non-obese women is their high hepatic-derived lipoprotein catabolic rate, which partly explains why they have lower plasma concentrations of apolipoprotein B-100 (Watts et al., 2000) and triglycerides (Mittendorfer et al., 2016). In addition to their high catabolic rate, women are also capable of secreting triglyceride-rich VLDLs when their liver is challenged with more fat (Hodson et al., 2015). Consequently, women are more effective than men in redirecting fat storage from liver to subcutaneous fat (Palmisano et al., 2018).

It can be concluded that women accumulate more fat in the subcutaneous depot because they have higher subcutaneous fat LPL activities and higher catabolic rate of hepatic-derived lipoproteins. The factors that allow men to accumulate more fat in the abdominal visceral depot will be discussed below.

### Dietary Fat Is Preferentially Stored as the Abdominal Visceral Fat by Men

The fat that is taken up by the adipocytes is primarily from lipoproteins, lipid particles with triglycerides in their core. The fact that men and women have different intestinal lipoproteins can potentially determine which body fat depot the dietary fat will be deposited to.

The organ that arguably secretes the most amount of fat is the small intestine, particularly during the postprandial state. Recall that the small intestine is surrounded by the abdominal visceral fat. Therefore, it is not surprising that the abdominal visceral fat can take up quite a significant amount of dietary fat from the intestinal lipoproteins. Studies have shown that about 21% of the ingested fat is stored in the intraperitoneal fat and about 6% of it is stored in the retroperitoneal fat by men (Marin et al., 1996). In contrast, only about 5% of the ingested fat is stored in the intraperitoneal fat by women (Votruba et al., 2007). These studies further support the notion that sex difference in regional body fat distribution is primarily determined by fat uptake rather than lipolysis.

### Men Produce Bigger and More Chylomicrons Than Women

To better understand why men’s abdominal visceral fat takes up more dietary fat than women’s, a closer look at the process of dietary fat absorption is necessary. Dietary fat is digested and absorbed by the small intestine. The absorbed dietary fat is secreted by the enterocytes in two major forms: chylomicrons (>80 nm in diameter) and VLDLs (30–80 nm).

Unlike VLDLs that can be produced during the preprandial states, the production of chylomicrons is primarily driven by dietary fat intake (Nauli et al., 2003, 2006, 2014; Drover et al., 2005). Importantly, when the small intestine is challenged with a higher amount of fat, it will produce bigger chylomicrons (Lo et al., 2008). These bigger chylomicrons tend to accumulate in the intestinal mucosa, as reflected by the higher recovery of the intraduodenally infused lipids in the intestinal mucosa, lower recovery in the lymph, and minimal recovery in the lumen at the end of the 6-h study. Since men generally consume a higher amount of dietary fat due to their higher energy intake (Wright and Wang, 2010), they are expected to produce bigger and more chylomicrons than women.

Studies comparing the postprandial chylomicrons in the plasma, in fact, indicate that chylomicrons transport significantly more dietary fat in men than in women (Knuth and Horowitz, 2006). The elevated plasma level of postprandial chylomicrons is also more prolonged in men than in women, suggesting further that it takes more time for men to transport bigger chylomicrons to the general circulation. Since the male participants were provided with more dietary fat in the studies, it remains to be determined if the reported effects were primarily due to their higher intake of fat. However, considering that men do normally have a higher dietary fat intake than women (Wright and Wang, 2010), these studies are still highly relevant physiologically. Of note, all of the female participants in the studies were in their follicular phase.

Since the serum level of estrogen is significantly higher than progesterone in the mid-to-late follicular phase, the estrogen effect on the size of chylomicrons warrants more investigation. Studies utilizing rodents indicate that even with a comparable amount of fat entering the lumen of the digestive tract, the chylomicrons produced by males transport more dietary fat than those produced by females; and the VLDLs produced by females transport more dietary fat than those produced by male (Vahouny et al., 1980).

From the animal to human studies discussed above, we can conclude that men transport more dietary fat through chylomicrons most likely because these chylomicrons are bigger and more than those of women. These sex differences in lipoprotein secretion can be further attributed to men’s higher intake of fat and possibly hormonal regulation.

### Chylomicrons May Preferentially Promote the Accumulation of the Abdominal Visceral Fat

There are several similarities and differences between chylomicrons and intestinal VLDLs. Regarding their similarities, both are apolipoprotein B-48-containing lipoproteins. They are also secreted to the capillary-rich lamina propria by the enterocytes (see Figure 1). However, their transport routes are quite different, which may determine the fat depot they will preferentially supply their dietary fat to.

Chylomicrons preferentially promote the accumulation of the abdominal visceral fat. High-fat meal, which triggers more chylomicron production, decreased the proportions of meal fat stored in the subcutaneous fat of both men and women (Votruba and Jensen, 2006). The unaccounted for meal fat in that study was likely stored in the abdominal visceral fat. It is important to note that the unaccounted for meal fat was found previously to be correlated with the amount of abdominal visceral fat (Romanski et al., 2000).

#### Chylomicrons Are Retained Longer in the Intestinal Mucosa

Since the lymphatic capillaries of the intestine allow bigger particles to enter their lumen relative to the blood capillaries, the lymphatic system is the predominant transport route for most of the chylomicrons. In addition to the transport by the lymphatics, VLDLs are capable of entering the lumen of the blood capillaries (Takahara et al., 2013). Particles as large as 30 nm in diameter have been shown to pass through the walls of the abdominal visceral blood capillaries (Simionescu et al., 1972). In fact, up to about 39% of the dietary triglycerides have been shown to be transported by the portal blood circulation (Mansbach et al., 1991; Mansbach and Dowell, 1993). Due to the fact that chylomicrons transport more dietary fat in males than in females, it is not surprising that males also have a higher lymphatic transport of dietary triglycerides than females (Vahouny et al., 1980).

Relative to the blood circulation, the lymphatic circulation is a low-pressure system that depends on the contraction of the surrounding muscles, such as the diaphragm and other abdominal muscles, to propel the lymph fluid. Consequently, the movement of the chylomicrons within the lamina propria and low-pressure lymphatics becomes a challenge during the postprandial state. This, again, is supported by the prolonged elevation of plasma chylomicrons in men (Knuth and Horowitz, 2006) as well as the higher recovery of the infused lipids in the intestinal mucosa when the intestine is challenged with more fat (Lo et al., 2008). Abdominal visceral fat accumulation has also been shown to correlate significantly with the delayed in postprandial lipid metabolism (Taira et al., 1999), supporting the notion that bigger chylomicrons that are retained longer in the intestinal mucosa promote the accumulation of the abdominal visceral fat.

Electron microscopy studies also show that during the postprandial state the lipoproteins in the lamina propria are bigger than those in the lymphatic lumen, further supporting the notion that chylomicrons do not enter the lymphatic lumen as readily as their smaller counterparts VLDLs (Takahara et al., 2013). Importantly, unlike the lumen of the blood capillaries, the lamina propria and the lymphatic lumen are visibly congested with chylomicrons.

#### Chylomicron Triglycerides Are Likely Susceptible to Hydrolysis in the Gut

The retention of chylomicrons in the lamina propria may predispose their triglycerides to the LPL hydrolysis. Although it has been reported that mesenteric fat expresses LPLs (Shimomura et al., 1993), it is still not known if the LPL activity in the abdominal visceral fat is different between men and women. As mentioned above, the LPL activities in the subcutaneous fat depots are generally higher in women, particularly in the lower part of the body (Arner et al., 1991; Votruba and Jensen, 2006).

LPLs can be tethered in the extracellular matrix by the HSPGs (Young et al., 2011). Therefore, it is tempting to speculate that relative to VLDLs, chylomicron triglycerides are more likely to be hydrolyzed by LPLs due to their higher retention in the lamina propria. In this regard, HSPGs may not simply serve as LPL reservoir for GPIHBP1 tethering on the capillary endothelia but may directly facilitate the LPL hydrolysis of the chylomicron triglycerides.

Alternatively, the LPLs that are present in the lymph (Huang et al., 1990; Qin et al., 2011) may also hydrolyze the chylomicron triglycerides. Angptl4, which is secreted by adipocytes and liver during fasting (Cushing et al., 2017), inhibits the LPLs that are not stabilized by GPIHBP1 (Sonnenburg et al., 2009; Lichtenstein et al., 2010). This autocrine/paracrine inhibition prevents fat accumulation by adipocytes during fasting. As the Angptl4 expression is reduced during postprandial state, the chylomicron triglycerides can then be potentially hydrolyzed by the disinhibited LPLs in the lymph.

Interestingly, the LPL expression in the mesenteric fat can be reduced by exercise (Shimomura et al., 1993). This suggests that exercise does not simply increase the energy expenditure but may also inhibit the dietary fat uptake by the abdominal visceral fat. Additionally, exercise may help reduce the retention of the chylomicrons in the intestinal mucosa (Havas et al., 1997, 2000). Contraction of the surrounding muscles during exercise should help propel the congested chylomicrons into the lymph and reduce further the susceptibility of their triglycerides to LPL hydrolysis.

#### Uptake of the Liberated Fatty Acids by the Abdominal Visceral Adipocytes

The liberated fatty acids that are the products of the LPL hydrolysis will then be delivered to the mesenteries either through the veins or lymphatics within the mesenteries (see Figure 1). Note that these veins and lymphatics are the convergences of the blood and lymphatic capillaries of the lamina propria, respectively. Since these veins and lymphatics are surrounded by the abdominal visceral adipocytes, the liberated fatty acids supply the abdominal visceral adipocytes prior to the subcutaneous adipocytes. Consequently, the LPL hydrolysis of the chylomicron triglycerides in the lamina propria should preferentially lead to the accumulation of abdominal visceral fat instead of subcutaneous fat. The transport of the liberated fatty acids by the veins within the mesenteries is supported by the fact that the unesterified fatty acid concentration is elevated in the portal vein after a bolus feeding of triglycerides (Kristensen et al., 2006). Of note, the hepatic portal vein receives blood from the mesenteries before draining them into the systemic blood circulation.

#### Leakiness of the Visceral Lymphatics Promotes the Abdominal Visceral Fat Accumulation

The idea that chylomicron retention in the extracellular matrix of the intestine can lead to the abdominal visceral fat accumulation is supported by the *Prox1* haploinsufficiency studies (Harvey et al., 2005). The studies show that *Prox1* ± mice, which have very leaky visceral lymphatics, accumulate a significant amount of abdominal visceral fat such that they develop obesity in their adulthood. The leaky intestinal lymphatics conceivably allow more chylomicrons to leave the lymphatic lumen and be retained in the extracellular matrix. Their enormous size may further prolong their retention, allowing their dietary fat to be hydrolyzed and stored by the surrounding abdominal visceral adipocytes. A recent study indicates that repairing the leaky lymphatics in *Prox1* ± mice prevents them from becoming obese, further confirming that the leaky lymphatics are responsible for their obesity phenotype (Escobedo et al., 2016). The study also suggests that the free fatty acids that are presumably liberated from the hydrolysis of chylomicron triglycerides may serve as the inducer of adipogenesis.

Lymphatic leakiness, which can be reduced by exercise (Hespe et al., 2016), is a normal part of aging (Zolla et al., 2015). The leakiness of the aging lymphatics may explain why the sex differences in the abdominal visceral fat accumulation become less pronounced with aging (Camhi et al., 2011) as well as the sex differences in lipid metabolism cannot all be attributed to sex hormones (Wang et al., 2011). It is becoming more apparent now that the roles of lymphatics in the development of android obesity cannot be disregarded.

**FIGURE 3:**
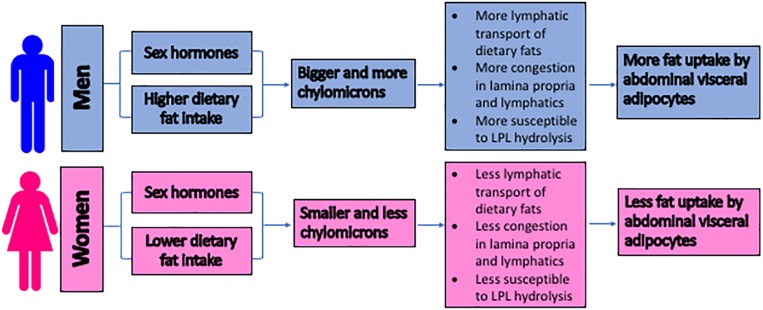
The proposed mechanisms for the sex differences in the development of the abdominal visceral fat. The size of lipoproteins is proposed to serve as an important factor in regulating the abdominal visceral adiposity. Due to the higher intake of dietary triglycerides and the potential hormonal regulation, men produce bigger and more chylomicrons. These chylomicrons trigger congestion within the lamina propria and lymphatics, subjecting their triglycerides to LPL hydrolysis. The subsequent uptake of the hydrolysis products by the surrounding adipocytes results in the accumulation of the abdominal visceral fat. LPL = lipoprotein lipase.

Figure 3 summarizes our proposed mechanisms of why men are more likely to develop abdominal visceral fat than pre-menopausal women. Note that the aging and exercise effects are not depicted in the figure.

## Conclusion

Of all of the factors contributing to the accumulation of abdominal visceral fat, lifestyle is arguably the most important. Based on our proposed mechanisms, spreading out the amount of dietary fat intake into several smaller meals should reduce the likelihood of abdominal visceral fat accumulation by reducing both the size and number of chylomicrons. Reducing the lipid load to the small intestine is also beneficial to the functions of the collecting lymphatics as high lipid load reduces their contraction frequency and amplitude (Kassis et al., 2016). By maintaining the lymphatic contraction, smaller meals may reduce the retention time of the chylomicrons in the lamina propria. This would consequently reduce the likelihood of abdominal visceral fat accumulation. In overweight minority youth studies, the higher calorie-consuming nibblers, indeed, accumulate less abdominal visceral fat than the lower calorie-consuming gobblers (House et al., 2014). The studies also take gender into account, that is, males are more likely to gobble and accumulate abdominal visceral fat than females.

Another important aspect of lifestyle is exercise. Besides increasing the energy expenditure, exercise may slow down the accumulation of abdominal visceral fat by increasing the flow of the chylomicrons within the lamina propria and lymphatics as well as reducing both the LPL expression in the mesenteric fat and the leakiness of the lymphatics.

There are other potential factors that may contribute to the development of abdominal visceral fat. But considering that it is a strong independent predictor of mortality, understanding the mechanisms of its development is critical. Based on our proposed mechanisms, exercising and eating a diet low in fat—or at least spreading the fat intake into several smaller meals—should help in slowing down the development of abdominal visceral fat.

## Data Availability Statement

The data supporting the conclusions of this article are from previously published articles. Please refer to the reference section of this article.

## Author Contributions

AN wrote the manuscript. SM generated the figures.

## Conflict of Interest

The authors declare that the research was conducted in the absence of any commercial or financial relationships that could be construed as a potential conflict of interest.

## Abbreviations

Angptl4: angiopoietin-like 4
BMI: body mass index
GPIHBP1: glycosylphosphatidylinositol-anchored high density lipoprotein-binding protein 1
HSPGs: heparin sulfate proteoglycans
LPL: lipoprotein lipase
VLDL: very low density lipoprotein.
